# Celery and Spinach Flavonoid-Rich Extracts Enhance Phytoalexin Production in Powdery Mildew-Infected Cucumber Leaves

**DOI:** 10.3390/plants14152414

**Published:** 2025-08-04

**Authors:** Hajar Soleimani, Shima Gharibi, Santa Olga Cacciola, Reza Mostowfizadeh-Ghalamfarsa

**Affiliations:** 1 Department of Plant Protection, School of Agriculture, Shiraz University, Shiraz 71441-13131, Iran; hajarsoleimani97@gmail.com; 2 Core Research Facilities, Isfahan University of Medical Sciences, Isfahan 81746-73461, Iran; s.gharibi@mail.mui.ac.ir; 3 Department of Agriculture, Food and Environment, University of Catania, Via S. Sofia 100, 95123 Catania, Italy

**Keywords:** *Apium graveolens*, *Spinacia oleracea*, *Cucumis sativus*, flavonoid-rich plant extracts, phenolic compounds, phytoalexins, powdery mildew

## Abstract

Phytoalexins are antimicrobial compounds of diverse chemical classes whose production is triggered in plants in response to pathogen infection. This study demonstrated that spraying with a celery flavonoid-rich extract (CFRE) or a spinach flavonoid-rich extract (SFRE) enhanced the production of phytoalexins in cucumber leaves artificially infected with powdery mildew incited by *Podosphaera fusca*. High-performance liquid chromatographic (HPLC) analysis revealed a noticeable increase in the content of phenolic acids, including caffeic acid, ellagic acid, ferulic acid, gallic acid, *p*-coumaric acid, and syringic acid, as well as the flavonoid rutin in both non-inoculated and inoculated leaves of cucumber seedlings treated with CFRE and SFRE, compared to healthy untreated leaves used as a control. Fluorescence microscopy revealed the accumulation of phenolic acid compounds in chloroplasts and at the periphery of epidermal cells. Overall, results suggest the reduced severity of *P. fusca* infection following the application of CFRE and SFRE in cucumber leaves could be due, at least in part, to the production of phytoalexins of polyphenolic nature. These findings provide insights into the mechanisms of systemic resistance induced by CFRE and SFRE. Moreover, they confirm these two natural flavonoid-rich products could be promising alternatives to synthetic chemical fungicides for the safe and ecofriendly control of cucumber powdery mildew.

## 1. Introduction

*Podosphaera fusca* (Fr.) U. Braun & Shishkoff (Syn. *Podosphaera xanthii* (Castagne) U. Braun & Shishkoff) is an obligate biotrophic pathogen responsible for the powdery mildew of cucumber (*Cucumis sativus* L.). This disease poses a significant threat to cucumber in various geographical regions and is particularly severe in greenhouse crops [1]. It infects leaves, stems, and fruits and spreads rapidly in cucumber plantings [2]. Although chemical fungicides are widely used for the management of cucumber powdery mildew, their application has been limited due to concerns about human health and environmental impact [1,3]. Consequently, research efforts have focused on environmentally safe alternatives, such as bioactive plant extracts [3,4]. Utilizing plant protection strategies based on these natural products benefits human health, reduces environmental pollution, prevents the risk of pathogen resistance, and is compatible with organic production systems.

Plant extracts with antifungal activity have been extensively used for controlling fungal plant diseases [5,6,7,8,9]. A conspicuous body of evidence indicates that, in treated plants, these natural products induce systemic resistance against pathogens [8,9,10,11,12,13,14,15].

Plants exhibit a range of molecular and biochemical defense mechanisms in response to biotic and abiotic stresses, such as infections by viral, bacterial, fungal, and oomycete pathogens, drought, and salinity [16,17,18]. These defense mechanisms are often induced by elicitors, which prime plant defense responses, such as the synthesis and accumulation of phenolic compounds and the upregulation of PAMP-triggered immunity (PTI), which refers to the immune response activated by pathogen-associated molecular patterns (PAMPs) [19,20]. The accumulation of secondary metabolites within plant tissues contributes to the formation of biochemical and physical barriers against pathogens. For instance, the accumulation of phenols contributes to enhancing resistance against fungal pathogens by strengthening the cell walls through processes of lignification and suberization [21,22,23]. Several genes encode enzymes involved in the biosynthetic pathways of these secondary metabolites, as in the case of the phenylpropanoid pathway, which leads to the accumulation of secondary metabolites, including compounds such as flavonoids and phenolic acids, in some cases acting as phytoalexins [17,24]. Key enzymes in this pathway include phenylalanine ammonia-lyase, polyphenol oxidase, 4-coumarate-CoA ligase, and chalcone, which play crucial roles in the biosynthesis of these phytoalexins and contribute to plant resistance mechanisms [25].

Phytoalexins are defined as low-molecular-weight secondary metabolites, a chemically heterogeneous group of antimicrobial substances produced de novo and in appreciable amounts by plants in response to pathogen attacks. In a broader sense, the term phytoalexins also includes those antimicrobial secondary metabolites inherently present in the plant, whose concentration increases as a consequence of pathogen attack [25]. Phytoalexins comprise diverse classes of chemical compounds, such as polyphenols and terpenoids, and may be produced specifically by a plant family, as in the case of rishtin, or even by a single plant variety, as in the case of essential oils [26,27]. Most phytoalexins, such as polyphenols, have antioxidant properties. These compounds are characterized by a broad-spectrum antimicrobial efficacy, and most are synthesized via the shikimate pathway, resulting in the production of distinct structural classes of isoflavonoids and phenylalanine [28]. The synthesis of phytoalexins is regulated by specific biosynthetic enzymes, such as phenylalanine ammonia-lyase, which are activated in response to both biotic and abiotic stresses. Phytoalexins have been shown to play a role in the defense of cucumber against powdery mildew. The application of a *Reynoutria sachalinensis* L. extract on cucumber leaves infected by powdery mildew induced resistance to the disease and enhanced the activity of chalcone synthase and the production of flavonoids [19]. Daayf et al. [29] reported that the treatment of cucumber leaves infected with *P. fusca* with *R. sachalinensis* extract induced the production of caffeic acid, ferulic acid, and *p*-coumaric acid. Certain phenolic acids and flavonoids that accumulate post-infection and post-treatment with plant extracts, which demonstrate antimicrobial activity, may be regarded as phytoalexins [19,29].

Recent studies demonstrated that foliar sprays with celery flavonoid-rich extract (CFRE) and spinach flavonoid-rich extract (SFRE) effectively reduced the severity of powdery mildew in *P. fusca*-inoculated cucumber seedlings [9,30]. CFRE and SFRE were found to elicit multiple defense responses in cucumber leaves, as evidenced by the enhanced activity of defense-related enzymes, including β-1.3-glucanase, chitinase, peroxidase, phenyl alaninammonia-lyase, and polyphenol oxidase, and the overexpression of the corresponding encoding genes. Moreover, the application of CFRE and SFRE led to an increase in chlorophyll content and an accumulation of total phenolic compounds. There was a consistent positive correlation between total phenolic compounds, the activities of defense-related enzymes, and the expression of respective encoding genes. Based on these findings, it has been hypothesized that cucumber leaves inoculated with *P. fusca* and treated with either CFRE or SFRE phenolic compounds act as phytoalexins. The objectives of this study were as follows: i. To identify the phenolic compounds whose production in cucumber leaves is enhanced by the application of CFRE and SFRE. ii. To investigate the time course of the accumulation of these putative phytoalexins in cucumber leaves after the application of CFRE and SFR. iii. To localize the cellular sites where the phenol class accumulates following the application of CFRE and SFRE.

## 2. Results

### 2.1. Chemical Analysis of Phytoalexins in Cucumber Leaves Induced by CFRE

The HPLC chromatogram of phenolic acids and flavonoids identified in the leaves of both inoculated and non-inoculated cucumber seedlings after treatment with CFRE is shown in Figure S1. Two-way ANOVA revealed that the CFRE foliar treatment significantly (*p* ≤ 0.001) improved phytoalexin concentration in both non-inoculated and inoculated cucumber leaves compared to their respective water-treated controls (Table S1). Furthermore, the ANOVA results indicated that the sampling time intervals after the treatment and the interaction between the four diverse treatment sets and sampling time intervals had a significant (*p* ≤ 0.001) effect on the concentration of phytoalexins in the cucumber leaves (Table S1). The effect was significant for ferulic acid at *p* ≤ 0.01 and for *p*-coumaric acid and syringic acid at *p* ≤ 0.05.

#### 2.1.1. Analysis of the Concentration of Phenolic Acid Compounds in Cucumber Leaves

##### Caffeic Acid

**Figure 1:**
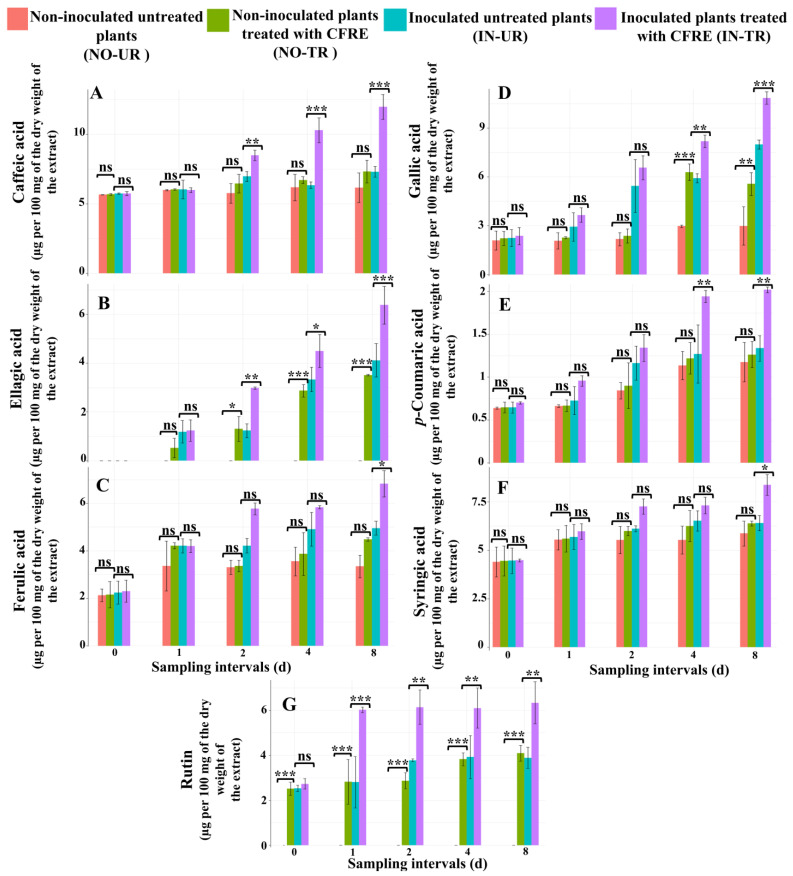
Measured phytoalexin amounts (µg per 100 mg of the dry weight of the extract) (**A**) caffeic acid; (**B**) ellagic acid; (**C**) ferulic acid; (**D**) gallic acid; (**E**) *p*-coumaric acid; (**F**) syringic acid; and (**G**) rutin in the inoculated/non-inoculated cucumber leaves at different sampling intervals (0, 1, 2, 4, and 8 d) following treatment with celery flavonoid-rich extract (CFRE). Significant differences between each group and its respective control at the same time interval are indicated by symbols (Tukey’s multiple comparison test): 0 ‘***’ for *p* ≤ 0.001, ‘**’ for *p* ≤ 0.01, ‘*’ for *p* ≤ 0.05, and ns = non-significant.

Caffeic acid concentrations did not differ significantly between healthy CFRE-treated (NO-TR) and untreated (NO-UR) cucumber seedlings at any sampling time interval (Figure 1A). By contrast, in inoculated seedlings treated with CFRE (IN-TR), caffeic acid concentrations significantly increased compared to inoculated untreated (IN-UR) seedlings on day two, day four, and day eight after CFRE treatment (*p* ≤ 0.01 on d 2, *p* ≤ 0.001 on d 4 and d 8; Figure 1A).

##### Ellagic Acid

No ellagic acid was detected in any of the four sets of cucumber seedlings day zero following the application of CFRE. The ellagic acid concentration in the NO-TR seedlings exhibited a significant rise on day two, day four, and day eight following the CFRE application (*p* ≤ 0.05 on d 2 and *p* ≤ 0.001 on d 4 and d 8; Figure 1B), which was different from the NO-UR seedlings. In IN-TR seedlings, ellagic acid levels significantly increased on day two, day four, and day eight after CFRE application (*p* ≤ 0.01, *p* ≤ 0.05, and *p* ≤ 0.001, respectively), compared to IN-UR plants (Figure 1B).

##### Ferulic Acid

The variation in ferulic acid concentration across all sampling time intervals in the NO-TR cucumber seedlings was not significant compared to the NO-UR seedlings (Figure 1C). The same results were observed for the inoculated seedlings. However, on day eight, following the application of CFRE, the concentration of ferulic acid in the IN-TR seedlings was significantly higher (*p* ≤ 0.05) compared to the IN-UR seedlings (Figure 1C).

##### Gallic Acid

Gallic acid levels in NO-TR cucumber seedlings significantly increased on day four (*p* ≤ 0.001) and day eight (*p* ≤ 0.01) after CFRE application compared to NO-UR seedlings (Figure 1D). Similarly, gallic acid concentrations in IN-TR seedlings significantly increased on day four (*p* ≤ 0.01) and day eight (*p* ≤ 0.001) compared to IN-UR seedlings (Figure 1D).

##### *p*-Coumaric Acid

*p*-coumaric acid concentrations in NO-TR cucumber seedlings did not significantly differ from NO-UR seedlings at any time interval (Figure 1E). By contrast, *p*-coumaric acid concentrations in IN-TR plants significantly increased on day four and day eight after CFRE application (*p* ≤ 0.01) compared to IN-UR seedlings (Figure 1E).

##### Syringic Acid

Syringic acid concentrations in NO-TR cucumber seedlings did not significantly differ from NO-UR plants at any time interval (Figure 1F). In IN-TR seedlings, syringic acid concentrations significantly increased on day eight after CFRE application (*p* ≤ 0.05) compared to IN-UR seedling leaves (Figure 1F).

#### 2.1.2. Analysis of the Concentration of Flavonoid Compounds in Cucumber Leaves

##### Luteolin, Quercetin, and Rutin

Results showed that in the four seedling sets, the two flavonoid compounds luteolin and quercetin were not detected. In NO-TR cucumber seedlings, rutin concentration significantly increased at all time intervals (*p* ≤ 0.001) following CFRE application compared to NO-UR seedling leaves (Figure 1G). Similarly, in IN-TR plants, CFRE treatment significantly increased rutin concentration on day one (*p* ≤ 0.001), day two (*p* ≤ 0.01), day four (*p* ≤ 0.01), and day eight (*p* ≤ 0.01) post-spraying compared to IN-UR seedlings (Figure 1G).

### 2.2. Heatmap of Phytoalexins in Cucumber Leaves Induced by CFRE

**Figure 2:**
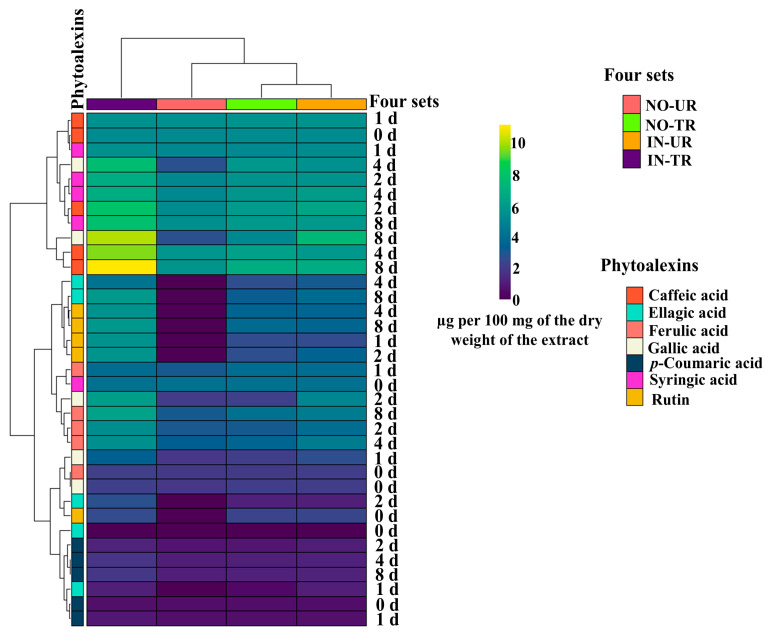
Clustering heatmap of the measured phytoalexin amounts (caffeic acid, ellagic acid, ferulic acid, gallic acid, *p*-coumaric acid, rutin, and syringic acid) after foliar application of celery flavonoid-rich extract in the four sets of cucumber seedlings: inoculated untreated seedlings (IN-UT), inoculated treated seedlings (IN-TR), non-inoculated untreated seedlings (NO-UT), and non-inoculated treated seedlings (NO-TR), at various sampling time intervals (0, 1, 2, 4, and 8 d).

The phenolic compounds, including five phenolic acids and one flavonoid compound produced across the four cucumber seedling sets examined, were divided into three major clusters: the first cluster included caffeic acid, gallic acid, and syringic acid; the second cluster included ellagic acid, ferulic caid, gallic acid, syringic acid, and rutin; the third cluster included ellagic acid, ferulic acid, gallic acid, *p*-coumaric acid, and rutin (Figure 2). The results in Figure 2 also show that the caffeic acid and gallic acid concentrations in the IN-UR and IN-TR cucumber seedlings after CFRE application were higher than those of the other compounds. Additionally, the caffeic acid concentration in the NO-TR cucumber seedlings was higher than that of the other phytoalexins.

### 2.3. Chemical Analysis of Phytoalexins in Cucumber Leaves Induced by SFRE

The HPLC chromatogram displaying the identified phenolic acid and flavonoid compounds in both inoculated and non-inoculated seedlings following the SFRE treatment is shown in Figure S2. Results from the two-way ANOVA analysis revealed that the SFRE treatment significantly affected the concentrations of caffeic acid, ellagic acid, ferulic acid, gallic acid, *p*-coumaric acid, syringic acid, and rutin in the leaves of treated cucumber seedlings, including both inoculated (IN-TR) and non-inoculated (NO-TR) seedlings, compared to the respective water-treated controls (IN-UR and NO-UR) (Table S2). The ANOVA results indicated that sampling times and the two-way interactions between the four treatment sets and sampling times on the accumulation of phytoalexins were significant (*p* ≤ 0.001), except for ferulic acid, *p*-coumaric acid, rutin, and syringic acid, which were significant at *p* ≤ 0.01 (Table S2).

#### 2.3.1. Analysis of the Concentration of Phenolic Acid Compounds in Cucumber Leaves

##### Caffeic Acid

**Figure 3:**
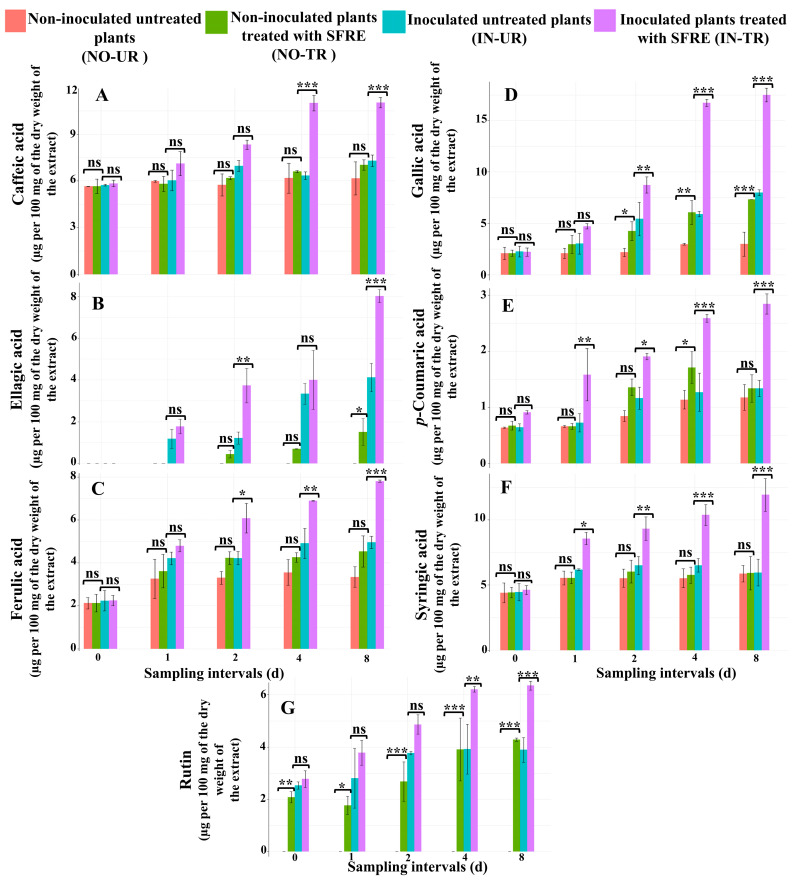
Measured phytoalexin amounts (µg per 100 mg of the dry weight of the extract) (**A**) caffeic acid; (**B**) ellagic acid; (**C**) ferulic acid; (**D**) gallic acid; (**E**) *p*-coumaric acid; (**F**) syringic acid; and (**G**) rutin in the non-inoculated/inoculated cucumber leaves at different sampling intervals (0, 1, 2, 4, and 8 d) following treatment with spinach flavonoid-rich extract (SFRE). Significant differences between each group and its respective control at the same time interval are indicated by symbols (Tukey’s multiple comparison test): 0 ‘***’ for *p* ≤ 0.001, ‘**’ for *p* ≤ 0.01, ‘*’ for *p* ≤ 0.05, and ns = non-significant.

In the non-inoculated SFRE-treated cucumber seedlings, the caffeic acid concentration exhibited no significant changes across all sampling time intervals in comparison to the NO-UR seedlings (Figure 3A). On day four and day eight post-application of SFRE, in the IN-TR seedlings, there was a significant increase in caffeic acid levels at *p* ≤ 0.001 compared to the IN-UR water-treated seedlings used as a control (Figure 3A).

##### Ellagic Acid

The ellagic acid concentration at a single sampling time point, taken eight days after the application of SFRE, was significantly higher (*p* ≤ 0.05) in the NO-TR cucumber seedlings compared to the NO-UR seedlings (Figure 3B). Additionally, on day two and day eight after the treatment with SFRE, the ellagic acid concentration significantly increased (*p* ≤ 0.01 and *p* ≤ 0.001, respectively) in the IN-TR cucumber seedlings compared to the IN-UR seedlings (Figure 3B).

##### Ferulic Acid

In NO-TR cucumber seedlings, after SFRE treatment, the level of ferulic acid did not show significant variation at any time intervals compared to the NO-UR seedlings (Figure 3C). In comparison to the IN-UR seedlings, the ferulic acid concentration significantly increased on day two (*p* ≤ 0.05), day four (*p* ≤ 0.01), and day eight (*p* ≤ 0.001) after the treatment with SFRE in IN-TR seedlings (Figure 3C).

##### Gallic Acid

In NO-TR cucumber seedlings, gallic acid levels significantly increased on day two (*p* ≤ 0.05), day four (*p* ≤ 0.01), and day eight (*p* ≤ 0.001) after SFRE application compared to NO-UR seedlings (Figure 3D). In IN-TR seedling leaves, gallic acid concentrations significantly increased on day two (*p* ≤ 0.01), day four (*p* ≤ 0.001), and day eight (*p* ≤ 0.001) post-application of SFRE, compared to IN-UR seedlings (Figure 3D).

##### *p*-Coumaric Acid

In NO-TR cucumber seedlings, the *p*-coumaric acid concentration significantly increased on day four after SFRE treatment (*p* ≤ 0.01), compared to NO-UR seedlings (Figure 3E). In IN-TR seedlings, the *p*-coumaric acid concentration significantly increased on day one (*p* ≤ 0.01), day two (*p* ≤ 0.05), day four (*p* ≤ 0.001), and day eight (*p* ≤ 0.001) post-SFRE treatment, but did not increase significantly on day zero (Figure 3E). By contrast, in the leaves of IN-UR seedlings, the *p*-coumaric acid concentration was significantly higher compared to NO-UR seedlings.

##### Syringic Acid

In NO-TR seedlings, on day zero, day one, day two, day four, and day eight, syringic acid concentrations did not significantly differ from NO-UR seedlings (Figure 3F). By contrast, in the leaves of IN-TR seedlings, after SFRE treatment, syringic acid concentrations significantly increased compared to IN-UR seedlings (*p* ≤ 0.05, *p* ≤ 0.01, *p* ≤ 0.001, and *p* ≤ 0.001, on d 1, d 2, d 4, and d 8, respectively) (Figure 3F).

#### 2.3.2. Analysis of the Concentration of Flavonoid Compounds in Cucumber Leaves

##### Luteolin, Quercetin, and Rutin

Quercetin and luteolin were not detected in any of the four seedling sets. Similarly, rutin was not detected in the NO-UR seedling leaves, indicating that the production of this flavonoid was induced by either the treatment or the fungal infection. Indeed, in NO-TR seedlings at subsequent sampling times, the concentration of rutin was significantly higher than in NO-UR water-treated seedlings and progressively increased (*p* ≤ 0.01 on d 0, *p* ≤ 0.05 on d 1, *p* ≤ 0.001 on d 2, *p* ≤ 0.001 on d 4, *p* ≤ 0.001 on d 8) (Figure 3G). In the leaves of IN-TR seedlings, the rutin concentration on day four (*p* ≤ 0.01) and day eight (*p* ≤ 0.001) was significantly higher than in the leaves of IN-UR seedlings (Figure 3G).

### 2.4. Heatmap of Phytoalexins in Cucumber Leaves Induced by SFRE

**Figure 4:**
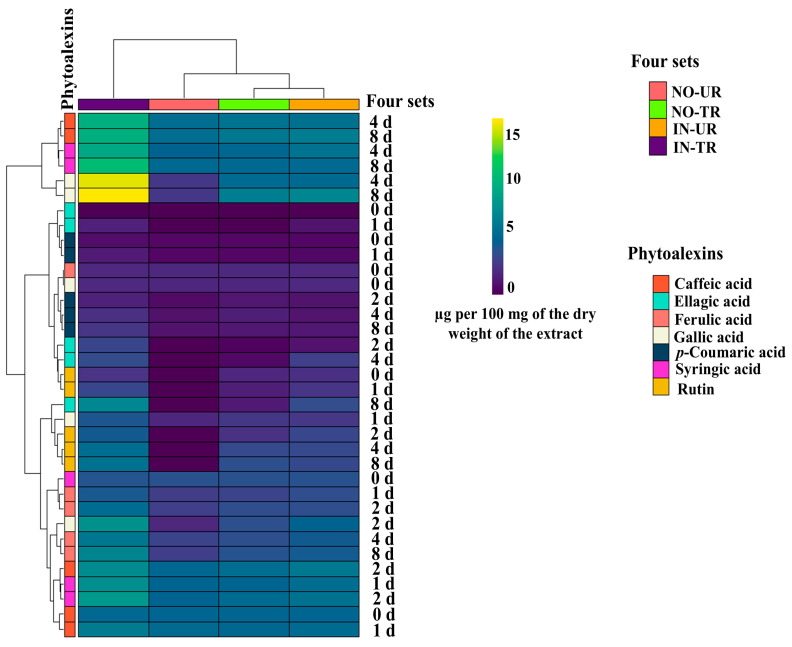
Heatmap of the measured phytoalexin amounts (caffeic acid, ellagic acid, ferulic acid, gallic acid, *p*-coumaric acid, rutin, and syringic acid) following foliar application of spinach flavonoid-rich extract in the four sets of cucumber seedlings: inoculated untreated seedlings (IN-UT), inoculated treated seedlings (IN-TR), non-inoculated untreated seedlings (NO-UT), and non-inoculated treated seedlings (NO-TR) at different sampling time intervals (0, 1, 2, 4, and 8 d).

The cluster analysis of phytoalexin accumulation induced by SFRE across the four seedling sets revealed that phenolic acids and rutin concentrations grouped into three major, distinct clusters. The first cluster comprised caffeic acid, gallic acid, and syringic acid; the second cluster comprised ellagic acid, ferulic acid, gallic acid, *p*-coumaric acid, and rutin; the third cluster comprised caffeic acid, ellagic acid, ferulic acid, gallic acid, syringic acid, and rutin. The heatmap (Figure 4) reveals that among the four sets of cucumber seedlings, the highest production of phytoalexins occurred in IN-TR seedlings. Phytoalexins produced at the highest concentrations included gallic acid, syringic acid, and caffeic acid, in that order. In IN-UR and NO-TR cucumber seedlings, the phytoalexins produced at the highest concentrations were caffeic acid and gallic acid.

### 2.5. Venn Diagram of Phytoalexins Induced by CFRE and SFRE Treatment in Cucumber Leaves

**Figure 5:**
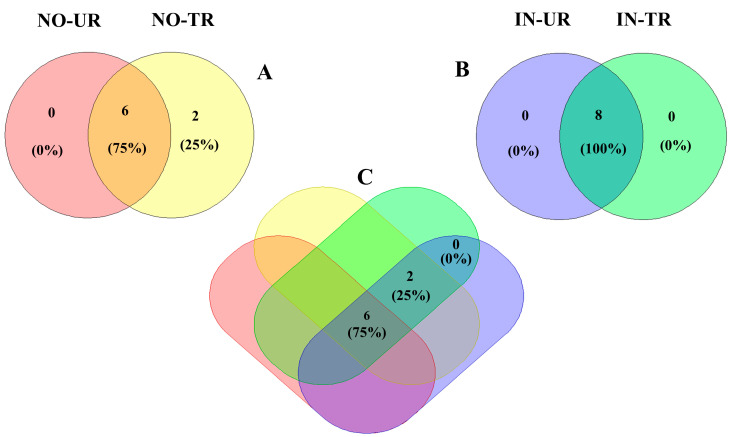
Venn diagrams showing the number and percentage of common phytoalexins (caffeic acid, ellagic acid, ferulic acid, gallic acid, *p*-coumaric acid, rutin, and syringic acid) present in and shared among (**A**): non-inoculated untreated seedlings (NO-UT) and non-inoculated treated seedlings (NO-TR); (**B**): inoculated untreated seedlings (IN-UT) and inoculated treated seedlings (IN-TR); (**C**): all four sets (non-inoculated untreated seedlings (NO-UT), non-inoculated treated seedlings (NO-TR), inoculated untreated seedlings (IN-UT), and inoculated treated seedlings (IN-TR)) of cucumber seedlings. Seedlings were sprayed with either celery or spinach flavonoid-rich extract, and samples were collected at various sampling time intervals after the treatment (0, 1, 2, 4, and 8 d).

The Venn diagram shows clearly that in the leaves of healthy seedlings, a phenolic acid compound (ellagic acid) and a flavonoid compound (rutin), which were not detected in the leaves of NO-UR seedlings, were produced de novo after the application of CFRE and SFRE (Figure 5A). All phytoalexins found in the leaves of powdery mildew-infected seedlings after CFRE and SFRE application (IN-TR seedlings) were also found in the leaves of untreated powdery mildew-infected seedlings (IN-UR seedlings) (Figure 5B). Finally, the Venn diagram (Figure 5C) shows that some phytoalexins (caffeic acid, ferulic acid, gallic acid, *p*-coumaric acid, and syringic acid) were present in the cucumber leaves of all four seedling sets and at all sampling times, while two compounds, the ellagic acid and rutin (Figure 1 and Figure 3), were not detected during the zero-day sampling time, indicating that they were not inherently present in the cucumber leaves and their production was primed by the treatment with flavonoid-rich plant extracts.

### 2.6. Fluorescence Microscopy

**Figure 6:**
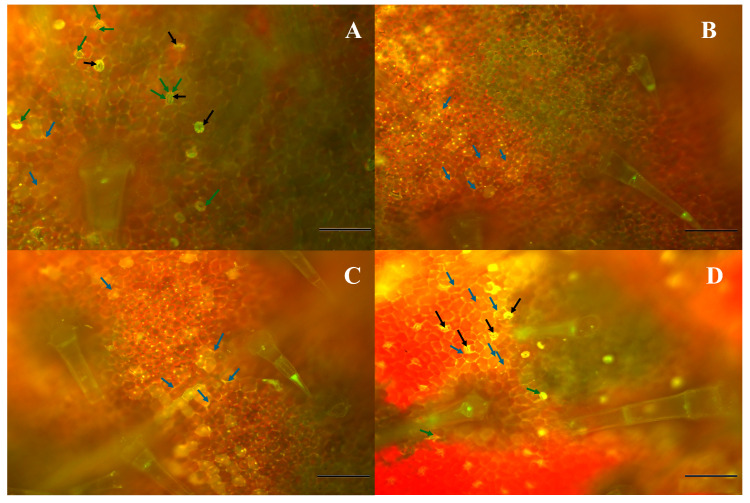
Fluorescence microscopy images showing the distribution patterns of phenolic acid compounds in inoculated cucumber leaves on 1 (**A**), 2 (**B**), 4 (**C**), and 8 (**D**) days post-treatment with celery flavonoid-rich extract. Black arrows = stomatal guard cells, blue arrows = periphery of epidermal cells, green arrows = chlorophyll. Bar = 10 μm.

**Figure 7:**
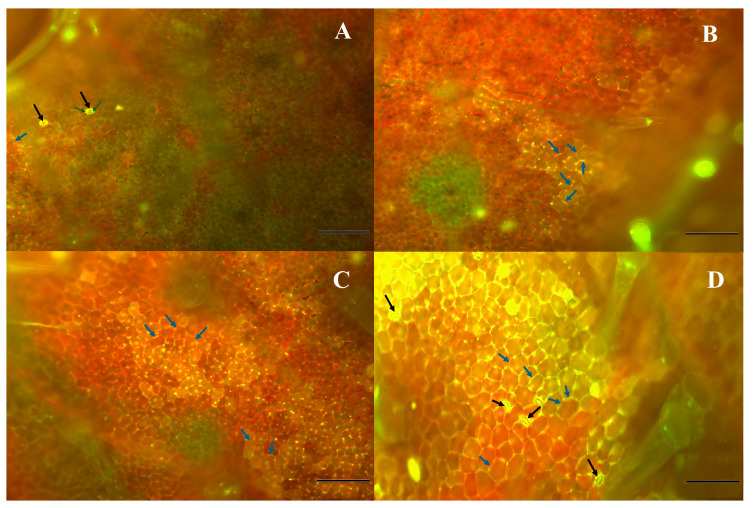
Fluorescence microscopy images showing the distribution patterns of phenolic acid compounds in inoculated cucumber leaves on 1 (**A**), 2 (**B**), 4 (**C**), and 8 (**D**) days post-treatment with spinach flavonoid-rich extract. Black arrows = stomatal guard cells, blue arrows = periphery of epidermal cells, green arrows = chlorophyll. Bar = 10 μm.

Figure 6 and Figure 7 show fluorescence microscopy images of phenolic acid compounds in the leaf tissues of *P. fusca*-inoculated cucumber seedlings treated with either CFRE or SFRE (IN-TR) at various time intervals following the treatment (days 1–8). The intensity of fluorescence indicative of phenolic acid compounds increased over the sampling period, reaching a peak on day eight. Phenolic acid compounds exhibiting a yellow fluorescence accumulated in stomatal guard cells and were associated with chloroplasts and cell walls. The accumulation of phenolic acid compounds around the periphery of epidermal cells was most likely associated with lignification and suberization of the cell wall. Chlorophyll red autofluorescence was also visible within the chloroplasts.

## 3. Discussion

Results of this study confirm that the production of antifungal, secondary, low-molecular-weight plant metabolites, such as phenolic acids and flavonoids, is part of a more complex defense response of cucumber plants triggered by the flavonoid-rich plant extracts’ CFRE and SFRE against cucumber powdery mildew caused by *P. fusca.*

Previous studies demonstrated that foliar spraying of CFRE and SFRE was effective in reducing the severity of powdery mildew and induced genetic and biochemical plant defense mechanisms against *P. fusca* in cucumber plants [9,30]. In particular, they showed that the treatment with CFRE or SFRE in powdery mildew-infected cucumber plants induced an increase in the concentration of total phenolic and flavonoid compounds in the leaves and a noticeable decrease in the severity of the disease. Since the antifungal activity of phenols is well known, it has been assumed that in powdery mildew-infected cucumber plants, these compounds act as phytoalexins. Phytoalexins play a crucial role in the defense mechanisms of plants [31,32]. They accumulate in plant tissues, inhibiting the growth of fungal pathogens. In particular, previous studies reported the production of phytoalexins as a response from plants to the infection of powdery mildews [33,34].

In the present study, phenolic compounds that accumulated in cucumber leaves, both as a response to powdery mildew infection and in treatment with flavonoid-rich plant extracts CFRE and SFRE, were identified as phenolic acid, caffeic acid, ellagic acid, ferulic acid, gallic acid, *p*-coumaric acid, and syringic acid, as well as the flavonoid compound rutin. All compounds were inherently present in the cucumber leaves, except for ellagic acid and rutin, which were produced de novo in plants infected with powdery mildew or treated with CFRE and SFRE. However, the production of all compounds was significantly enhanced by both the fungal infection and the treatment with flavonoid-rich plant extracts. Sampling at various time intervals after the inoculation of leaves with *P. fusca* and the application of CFRE or SFRE revealed that all phenols identified in this study accumulated progressively, and their concentrations peaked on the eighth day. The maximum concentration for all compounds was observed in the leaves of cucumber plants inoculated with *P. fusca* and treated with either CFRE or SFRE (here named set IN-TR), indicating a synergism between fungal infection and treatment with plant extracts in eliciting plant defense responses and providing strong evidence that CFRE and SFRE stimulate and enhance plant defense mechanisms. In cucumber seedlings of set IN-TR, the treatment with either CFRE or SFRE was very effective in controlling the powdery mildew, with a reduction of more than 90% compared to untreated seedlings, consistent with previous studies [9,30]. This confirms that the induction of plant resistance is the main action mechanism of flavonoid-rich plant extracts tested in this study and corroborates the hypothesis that cucumber plant phenolics act as phytoalexins against powdery mildew caused by *P. fusca*. Results of an HPLC analysis align, in part, with and complement those of previous studies reporting that *P. fusca* infection in cucumber leaves enhances the production of phytoalexins, including caffeic acid, ellagic acid, ferulic acid, gallic acid, syringic acid, *p*-coumaric acid, luteolin, and rutin [29,33,34]. The production of phenols acting as phytoalexins has also been observed in response to the infection of other plant pathogens. For instance, Li et al. [35] reported that caffeic acid concentration in tobacco plants increased following infection with *Ralstonia solanacearum* and promoted resistance against the pathogen. Moreover, other studies have also reported the accumulation of phytoalexins in plants following the application of plant extracts and natural compounds to control plant pathogens. For example, Margaritopoulou et al. [34] reported that the levels of caffeic acid, ferulic acid, *p*-coumaric acid, and syringic acid significantly increased in infected plants following the application of *R. sachalinensis* extract against cucumber powdery mildew. Similarly, Gao et al. [33] reported an increase in caffeic acid, chlorogenic acid, coumarin, ellagic acid, ferulic acid, gallic acid, luteolin, *p*-coumaric acid, quercetin, rutin, syringic acid, and vanillic acid in the cucumber leaves after treatment with ar-turmerone, a natural sesquiterpenic compound extracted from *Curcuma longa* L. and *C. zedoaria* (Christm.) Rosc., to control cucumber powdery mildew. Kaya et al. [36] found an increase in phenolic compounds after the application of essential oil compounds against *Botrytis cinerea* Pers. in *Vitis vinifera* L.

The enhanced production of phenolic acids and flavonoids in healthy plants after CFRE and SFRE treatment, compared to the healthy water-treated plants, is likely due to the accumulation of reactive oxygen species, which serve as signal molecules, a general defense response of plants to both biotic and abiotic stresses. Consistent with this finding, Gao et al. [33] observed increased levels of ellagic acid, gallic acid, *p*-coumaric acid, and rutin in healthy cucumber after treatment with ar-turmerone, while Daayf et al. [29] reported an enhancement in the concentration of caffeic acid and ferulic acid after treatment with *R. sachalinensis* extract.

The time course of the accumulation of single phenolic and flavonoid compounds in cucumber leaves as inferred from the HPLC analysis of leaves at diverse sampling times after the treatment with CFRE and SFRE, is consistent with the pattern of both enhanced activity of defense-related enzymes, β-1.3-glucanase, chitinase, peroxidase, phenyl alaninammonia-lyase and polyphenol oxidase and the overexpression of β-1,3-glucanase, chitinase, and phenylalanine ammonia-lyase genes reported in already published studies [9,30]. In particular, increasing concentrations of *p*-coumaric acid and other putative phytoalexins at various sampling time intervals after CFRE and SFRE application correlate with the upregulation of polyphenol oxidase, phenylalanine ammonia-lyase, and peroxidase enzymatic activities, as well as the corresponding gene expression. Furthermore, the increase in peroxidase and polyphenol oxidase activities might be linked to the generation of reactive oxygen species, which leads to the oxidation of phenolic compounds and their transformation into quinones. Also, our earlier research suggests that CFRE and SFRE treatments might trigger hormone-related signaling, particularly involving salicylic acid, in the induced resistance [9,30]. After treatment with CFRE and SFRE, the heightened activity of phenylalanine ammonia-lyase highlights its significance as a key enzyme in the phenylpropanoid pathway associated with salicylic acid biosynthesis. All these mechanisms contribute to systemic host plant resistance.

Fluorescence microscopy examination of phenolic acid compounds accumulation and localization in *P. fusca*-infected cucumber leaves treated with CFRE and SFRE confirms these compounds are involved in multiple resistance mechanisms induced by the treatment with bioactive plant extracts. Cucumber leaves were uniformly inoculated and treated with flavonoid-rich extracts. By the time of sampling, the fungal infection had spread, and the imaged regions included cells affected by the hypersensitive response. Plants are challenged by various biotic and abiotic stresses, which prompt them to generate reactive oxygen species (ROS), express defense-related genes, synthesize pathogenicity-related (PR) proteins, reinforce their cell walls locally, and produce phytoalexins. The presence of a fluorescent gradient indicates that the biosynthesis of phenolic acid compounds occurs primarily in the chloroplasts and guard cells, and then they are transported to other plant parts through the vascular tissue. It can be supposed that, along with the accumulation of phytoalexins, the cell wall fortification is a defense mechanism induced by the treatment with CFRE and SFRE. Phenylpropanoids and their derivatives form a wide group of compounds commonly secreted by plants and are involved in plant growth and development as well as in the defense against pathogens [37]. Phenylpropanoid derivatives are among the enhancers of plant cell wall fortification [23]. These compounds include coumarins, flavonoids, lignins, monolignols, phenylpropanoids, phenolic acids, and stilbenes, which are produced in the phenylpropanoid pathway [23,38]. The accumulation of phenylpropanoid derivatives within or at the periphery of cells, tissues, or organs may indicate the establishment of physical or chemical barriers that hinder the invasion by plant pathogens [38,39]. It is plausible that many of the phenolic compounds accumulate in vacuoles in glycosylated forms. Glycosylation enhances molecular stability and solubility upon pathogen challenges; deglycosylation may release active aglycones that contribute to defense responses. These processes could modulate both gene expression and local metabolite pools as part of a dynamic immune response. Results of fluorescence microscopy observations align with previous studies reporting the biosynthesis of phenolic acid compounds in chloroplasts and guard cells [40,41,42,43].

## 4. Materials and Methods

### 4.1. Extraction of Celery and Spinach Flavonoid-Rich Extract (CFRE, SFRE)

A batch of 100 g of either spinach or celery leaves was blended with 500 mL of ethanol (90%) (Merck, Darmstadt, Germany) and allowed to settle at room temperature for seven days. Then, the solvent was thoroughly removed using a rotary evaporator, resulting in around 7 g of dry extract. The dry extract was suspended in 40 mL of distilled water and 80 mL of chloroform (Merck) in a separatory funnel to extract chlorophyll, terpenoids, and fats. The aqueous layer above, which was abundant in flavonoid and phenolic compounds, was heated to 60 °C for one hour to ensure the evaporation of any residual chloroform. Once the sample had cooled to ambient temperature, it was freeze-dried using an F.D-V-450 freeze-dryer (Dorsatech, Tehran, Iran), weighed on a digital scale, and stored in a refrigerator for future use [9].

### 4.2. Effect of CFRE and SFRE Treatment on the Accumulation of Phytoalexins in Podosphaera Fusca-Infected Cucumber Leaves

#### 4.2.1. Plant Material and Fungal Inoculation

Cucumber seeds (var. ‘Super Dominus’ from the USA) were sown in plastic pots containing a 1:1:1 mixture of soil, sand, and peat moss. The pots were maintained under controlled conditions at 22 to 28 °C in the greenhouse. Relative humidity was maintained within the range of 70% to 80%, and a photoperiod of 14 h was arranged. Each experimental plot contained at least four uniform seedlings. Cucumber powdery mildew conidia were collected from infected plants and suspended in distilled water containing 1 mL L^−1^ Tween 20. Conidium concentration was determined using a hemacytometer and adjusted to 4 × 10^4^ conidia mL^−1^ [44]. This suspension was used to inoculate the plants by spraying. Powdery mildew spots became visible 8–10 d after inoculation.

#### 4.2.2. Experimental Design

Cucumber seedlings at the four-leaf stage were used to investigate the effect of CFRE and SFRE on the accumulation of phytoalexins in the leaves. For each of the two plant extracts, there were four sets of cucumber seedlings: 1. Set One: Seedlings inoculated with *P. fusca*, sprayed with 4 mg mL^−1^ of either CFRE or SFRE in distilled water (IN-TR seedlings), after the appearance of the first disease signs on the leaves according to the protocol of Soleimani et al. [9]. 2. Set Two: non-inoculated seedlings sprayed with 4 mg mL^−1^ of either CFRE or SFRE (NO-TR seedlings). 3. Set Three: Inoculated seedlings sprayed with distilled water right after the first disease signs appeared on the leaves (IN-UT seedlings). 4. Set Four, Non-inoculated seedlings sprayed with distilled water (NO-UT seedlings).

For each set, five pots were prepared, corresponding to five sampling times (0, 1, 2, 4, and 8 d). From each pot, three cucumber seedlings were selected, which served as biological replicates for that specific time point. Samples of both treated and untreated leaves were collected 0, 1, 2, 4, and 8 days after the application of CFRE and SFRE, then dried, and subsequently analyzed to assess the phytoalexin accumulation in the cucumber leaves.

### 4.3. Chemical Analysis of Phytoalexins in Cucumber Leaves

Phenolic compounds, including caffeic acid, ellagic acid, ferulic acid, gallic acid, luteolin, *p*-coumaric acid, quercetin, rutin, and syringic acid were quantified in the four sets of cucumber seedlings (treated/untreated with CFRE or SFRE) using an Agilent 1100 HPLC (High-Performance Liquid Chromatography) system (Azura, Mannheim, Germany) equipped with a diode array detector (260–370 nm) (Table S3). One g of each sample was dissolved in 10 mL of 80% methanol, and after filtering with a paper filter, it was dried. Following acid hydrolysis in 2N HCl at 95 °C, the hydrolyzed flavonoids from each dried sample were extracted using ethyl acetate. A 20 µL aliquot of the sample was dissolved in 1 mL of solvent B and injected into a Nova-Pak C18 column (4.6 × 250 mm, Waters, Milford, MA, USA), employing solvent A and solvent B at a flow rate of 0.8 mL min^−1^ with a gradient elution method. The solvents utilized for HPLC comprised 0.1 mL of formic acid and 99.9 mL of water as solvent A, and 99.9 mL of acetonitrile along with 0.1 mL of water as solvent B. The gradient program was set as follows: A: B (90:10) for 1 min, transitioning to 10–26% B over 40 min, then to 26–65% B over 30 min, and concluding with 65–100% B for 5 min. For the quantitative assessment of the aforementioned phenolic compounds, a standard curve was established using various concentrations of these standards (Sigma Aldrich, St. Louis, MO, USA). The experiment included three replicates, and the results were averaged and expressed as µg per 100 mg of the dry weight of the extract. The least squares method (R^2^ value) was employed to analyze the correlation between concentration and peak area of the standards [45].

### 4.4. Fluorescence Microscopy

To investigate the accumulation patterns of phenolic acid compounds in the leaves of cucumber seedlings, a fluorescence microscope, Olympus BX51 (Olympus, Tokyo, Japan), was used. Dried leaves were submerged in a reagent containing 2.52 mg mL^–1^ diphenyl boric acid 2-amino ethyl ester and 0.1% (*v*/*v*) Triton X-100 and incubated at room temperature for 2 h in the dark. To visualize phenolic acid compounds, WG (green) filters were used [46]. Diphenyl boric acid 2-amino ethyl ester is a fluorochrome that forms complexes with specific classes of flavonoids—particularly flavonols and phenolic compounds—resulting in characteristic yellow–orange fluorescence in plant tissue.

### 4.5. Statistical Analysis

For the greenhouse experiment, two-way ANOVA was applied to determine the effect of CFRE and SFRE on the accumulation of phytoalexin in the cucumber leaves of the four seedling sets (Inoculated untreated seedlings = IN-UT seedlings, inoculated treated seedlings = IN-TR seedlings, non-inoculated untreated seedlings = NO-UT seedlings, non-inoculated treated seedlings = NO-TR seedlings), at diverse sampling time intervals after the treatment. We employed Tukey’s multiple comparison for mean comparisons in the data analysis of phenolic compounds concentration (caffeic acid, ellagic acid, ferulic acid, gallic acid, *p*-coumaric acid, rutin, and syringic acid). Mean data for phenolic compounds concentrations were used to generate a heatmap. A Venn diagram was used to illustrate the number of phytoalexins produced after CFRE and SFRE treatment in four sets. Statistical analyses were performed using R software, version 4.2.3.

## 5. Conclusions

This study provided insights into the biochemical mechanisms of resistance induced by CFRE and SFRE applications in cucumber leaves against powdery mildew. Specifically, it highlighted a crucial role of the accumulation of phytoalexins belonging to the chemical classes of phenolic acids and flavonoids as part of the plant immune system activated by the treatment with plant extracts rich in flavonoids. This information is useful to better understand the mechanisms of action of these natural antifungal substances effective against cucumber powdery mildew. Although the findings are encouraging, further research is needed to implement the large-scale production of raw plant material for the extraction, to standardize industrial processing, to develop effective formulations of the extracts, and to optimize their application in greenhouses and open-air cultivations. Moreover, research should focus on evaluating the effectiveness of these flavonoid-rich plant extracts in controlling powdery mildew across different cucumber varieties and other cucurbit species. Upon determining the optimal formulation of these extracts as biofungicides, it is important to maintain a competitive production cost and to consider factors that ensure the longevity of the shelf-life of commercial products.

## Data Availability

The datasets generated during and analyzed during the current study are in Supplementary Tables or available from the corresponding author on reasonable request.

## Supplementary Materials

The following supporting information can be downloaded at https://www.mdpi.com/article/10.3390/plants14152414/s1, Figure S1: HPLC chromatograms of phytoalexin production in the non-inoculated (NO-TR) and inoculated (IN-TR) cucumber leaves at various sampling intervals (0, 1, 2, 4, and 8 d) after treatment with celery flavonoid-rich extract (CFRE); Figure S2: HPLC chromatograms of phytoalexin production in the non-inoculated (NO-TR) and inoculated (IN-TR) cucumber leaves at various sampling intervals (0, 1, 2, 4, and 8 d) after treatment with spinach flavonoid-rich extract (SFRE); Table S1: Two-way ANOVA analysis of the effect of celery flavonoid-rich extract (CFRE) at a concentration of 4 mg mL−1 on the accumulation of phytoalexins in cucumber leaves infected by powdery mildew, incited by Podosphaera fusca, at various sampling intervals (0, 1, 2, 4, and 8 d) following treatment with the CFRE; Table S2: Two-way ANOVA analysis of the effect of spinach flavonoid-rich extract (SFRE) at a concentration of 4 mg mL−1 on the accumulation of phytoalexins in cucumber leaves infected by powdery mildew incited by Podosphaera fusca, at various sampling intervals (0, 1, 2, 4, and 8 d) after the application of SFRE; Table S3: Rotation time and spectra absorption (nm) of compounds used in the high-performance liquid chromatography (HPLC) analysis.

## Abbreviations

The following abbreviations are used in this manuscript:

CFRE	Celery flavonoid-rich extract
SFRE	Spinach flavonoid-rich extract
IN-TR	Seedlings inoculated with *P. fusca*, sprayed with 4 mg mL^−1^ of either CFRE or SFRE in distilled water
NO-TR	Non-inoculated seedlings sprayed with 4 mg mL^−1^ of either CFRE or SFRE
IN-UT	Inoculated seedlings sprayed with distilled water
NO-UT	Non-inoculated seedlings sprayed with distilled water
PR	Pathogenicity-related
PTI	PAMP-triggered immunity
ROS	Reactive oxygen species
