# Supplementary material for: Celery and Spinach Flavonoid-Rich Extracts Enhance Phytoalexin Production in Powdery Mildew-Infected Cucumber Leaves
Source: Plants (Basel). 2025 Aug 4;14(15):2414. doi: 10.3390/plants14152414 (PMC12349301; doi:10.3390/plants14152414)
Supplement: Supplementary file 1 [file plants-14-02414-s001.zip › plants-3694753-supplementary.pdf]

Supplementary Table

Table S1. Two-way ANOVA analysis of the effect of celery flavonoid-rich extract (CFRE) at a concentration of 4 mg mL^{-1} on the accumulation of phytoalexins in cucumber leaves infected by powdery mildew, incited by *Podosphaera fusca*, at various sampling intervals (0, 1, 2, 4, and 8 d) following treatment with the CFRE.

Variables	DF	Phytoalexins													
		**Caffeic acid**		**Ellagic acid**		**Ferulic acid**		**Gallic acid**		***p*-Coumaric acid**		**Rutin**		**Syringic acid**	
		**MS**	**F**	**MS**	**F**	**MS**	**F**	**MS**	**F**	**MS**	**F**	**MS**	**F**	**MS**	**F**
Four sets	3	12.765***	40.592	15.661***	113.96	6.230***	26.485	27.205***	64.844	0.5145***	21.958	50.95 ***	165.235	3.041***	9.876
Sampling time	4	8.216 ***	26.126	16.236 ***	118.14	8.699***	36.981	31.205***	74.378	1.0346***	44.159	3.43***	11.117	6.482***	21.051
Four sets× Sampling time	12	2.724***	8.661	2.320 ***	16.88	0.788**	3.349	3.878***	9.244	0.0520*	2.218	1.04***	3.386	0.409*	1.329
Residuals	30	0.31		0.137		0.235		0.420		0.023		0.31		0.30	

Significant codes: 0 ‘***’ 0.001 ‘**’ 0.01 ‘*’ 0.05 ‘.’ 0.1 ‘ ’ 1; DF= Degree of freedom; F= F value; MS= Mean of squares.

Table S2. Two-way ANOVA analysis of the effect of spinach flavonoid-rich extract (SFRE) at a concentration of 4 mg mL^{-1} on the accumulation of phytoalexins in cucumber leaves infected by powdery mildew incited by *Podosphaera fusca*, at various sampling intervals (0, 1, 2, 4, and 8 d) after the application of SFRE.

Variables	DF	Phytoalexins													
		Caffeic acid		Ellagic acid		Ferulic acid		Gallic acid		*p*-Coumaric acid		Rutin		Syringic acid	
		MS	F	MS	F	MS	F	MS	F	MS	F	MS	F	MS	F
Four sets	3	15.357***	61.659	24.767***	113.58	10.772***	46.573	101.85***	191.63	2.3402***	64.310	40.65***	158.335	28.685***	51.868
Sampling time	4	6.356***	25.519	13.524***	62.03	11.149***	48.204	68.68***	129.22	1.5312***	42.078	5.22***	20.339	10.666***	19.287
Four sets× Sampling time	12	2.001***	8.034	3.657***	16.77	1.042**	4.505	15.88***	29.88	0.1475**	4.054	0.96**	3.756	2.402**	4.343
Residuals	30	0.249		0.218		0.231		0.53		0.0364		0.26		0.553	

Significant codes: 0 '***' 0.001 '**' 0.01 '*' 0.05 '.' 0.1 ' ' 1; DF= Degree of freedom; F= F value; MS= Mean of squares

Table S3. Rotation time and spectra absorption (nm) of compounds used in the high-performance liquid chromatography (HPLC) analysis.

Compound Type	Retention time	Spectra absorption (nm)
Flavonoids		
Luteolin	50.97	330
Quercetin	51.70	350
Rutin	30.64	330
Phenolic acids		
Caffeic acid	14.50	270
Ferulic acid	20.51	330
Ellagic acid	21.21	330
Gallic acid	5.40	270
p-Coumaric acid	27.03	330
Syringic acid	16.29	270

Supplementary Figures

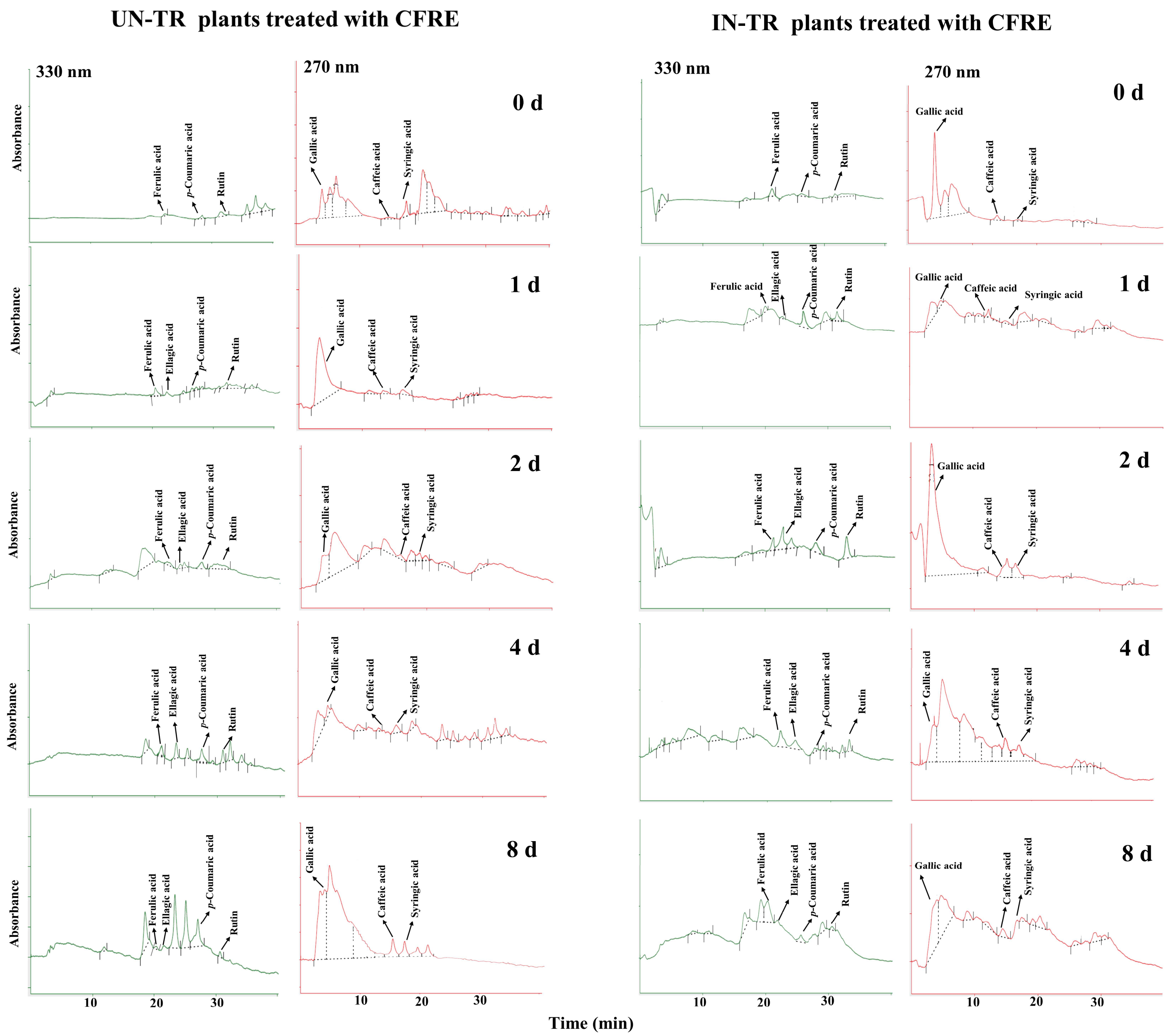

Figure S1. HPLC chromatograms of phytoalexin production in the non-inoculated (NO-TR) and inoculated (IN-TR) cucumber leaves at various sampling intervals (0, 1, 2, 4, and 8 d) after treatment with celery flavonoid-rich extract (CFRE).

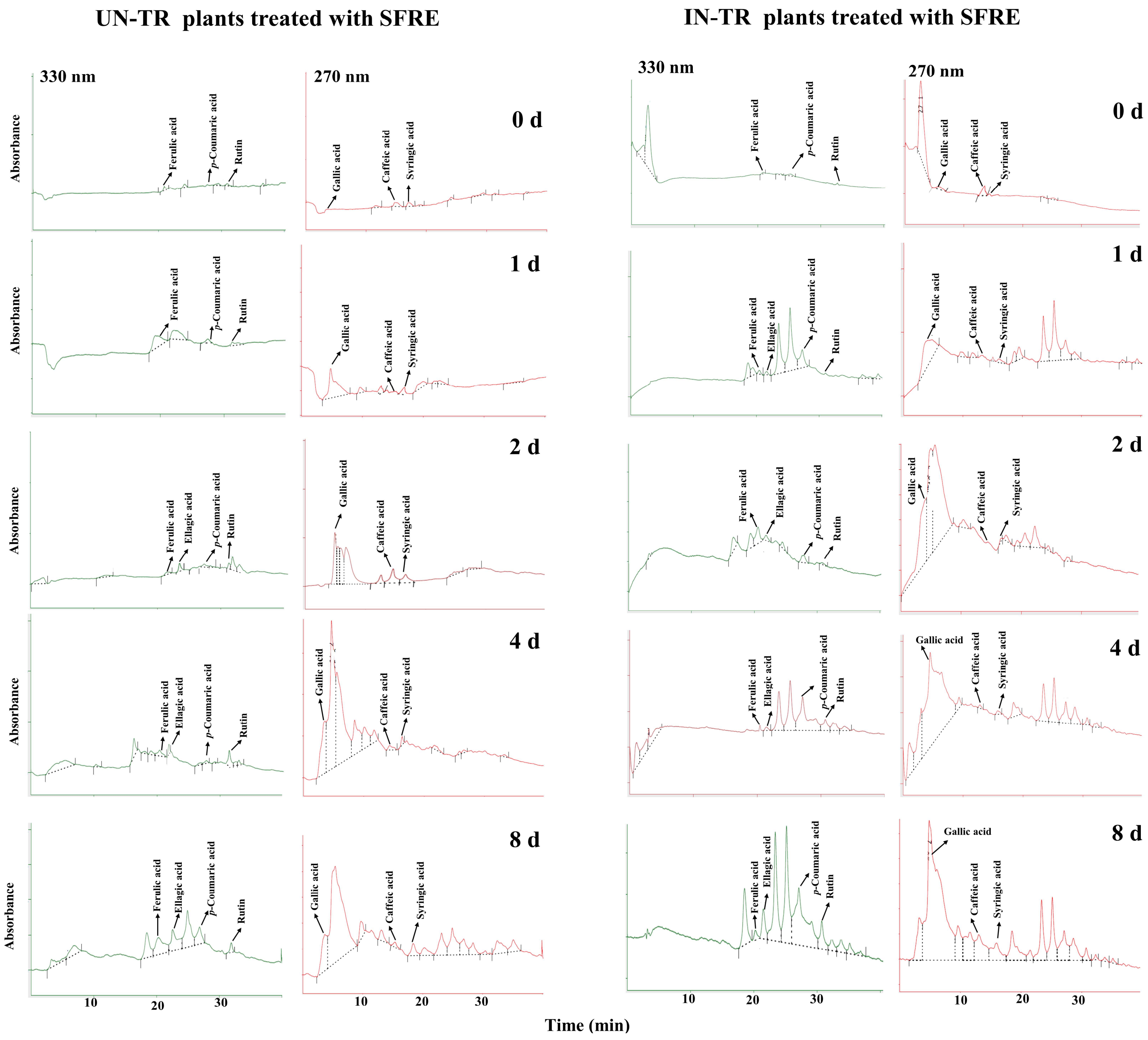

Figure S2. HPLC chromatograms of phytoalexin production in the non-inoculated (NO-TR) and inoculated (IN-TR) cucumber leaves at various sampling intervals (0, 1, 2, 4, and 8 d) after treatment with spinach flavonoid-rich extract (SFRE).
